# Rectovaginal fistula after low anterior resection in Chinese patients with colorectal cancer

**DOI:** 10.18632/oncotarget.17046

**Published:** 2017-04-11

**Authors:** Hongtu Zheng, Tianan Guo, Yuchen Wu, Cong Li, Sanjun Cai, Fangqi Liu, Ye Xu

**Affiliations:** ^1^ Department of Colorectal Surgery, Fudan University Shanghai Cancer Center, Shanghai 200032, China; ^2^ Department of Oncology, Shanghai Medical College, Fudan University, Shanghai 200032, China

**Keywords:** rectovaginal fistula, low anterior resection

## Abstract

Rectovaginal fistula is a postoperative complication of low anterior resection. We investigated the incidence of rectovaginal fistula (RVF) after low anterior resection, its risk factors and its optimal treatment. We analyzed data from 1,493 female patients who underwent low anterior resection for colorectal cancer between January 2006 and March 2016. We calculated the incidence of RVF and performed univariate and multivariate logistic regression analyses to identify risk factors. Twenty-four patients experienced RVF, giving an incidence of 1.61%. Univariate analysis revealed a short distance between the tumor and the anal verge (*p* < 0.001), longer surgery duration (*p* = 0.009), unsatisfactory anastomosis (*p* < 0.001), and greater intraoperative blood loss (*p* = 0.002) to be risk factors for RVF. Multivariate analysis showed that only distance between the tumor and the anal verge and unsatisfactory anastomosis were risk factors for RVF. Sixteen patients (66.7%) healed within a range of 30-1,225 days (median, 210 days). Twenty-one patients underwent surgery for diverting stoma; of those, 15 of them (71.4%) recovering after ostomy. These results indicate the primary risk factors for RVF are unsatisfactory anastomosis and short distance between the tumor and the anal verge. Most cases of RVF can be healed using a diverting stoma alone, without the need for additional surgery.

## INTRODUCTION

Rectovaginal fistula (RVF) refers to a pathologic hole formed from the neorectum to the vagina. Clinically, patients may present with feces or gas discharged from the vagina and inflammation in the pelvic cavity, which may lead to severe infection and poor quality of life. Although numerous studies have investigated RVF, very few reports have focused on RVF resulting from low anterior resection (LAR) of colorectal tumors. The incidence of post-LAR RVF reported in the literature is variable, ranging from 0.9% to 9.9% [1–7]. Because of limited research in this area and the small number of cases included in previous studies [6], the high-risk factors of post-LAR RVF remain unclear. Therefore, the incidence and risk factors of post-LAR RVF warrant further investigation.

As a postoperative complication of LAR, RVF accounts for only 5% of all anorectal fistulas [8]. However, once RVF occurs, it's difficult to treat [9]. As RVF has a significant impact on quality of life, physicians should ensure timely treatment to promote a fast recovery for patients with RVF. To date, there is no standard or widely accepted treatment for RVF [10, 11]. Patients who received surgical treatment are generally considered to have a better prognosis compared with those receiving conservative treatment [12]. However, whether transverse colostomy is superior to ileostomy to repair RVF, whether rectovaginal fistula need to be repaired, and what the optimal time is for stoma closure are still controversial and warrant further investigation.

In this study, we analyzed 24 cases of postoperative RVF out of 1,493 patients with colorectal cancer treated with LAR at Fudan University Shanghai Cancer Center between January 2006 and March 2016. We aimed to quantify the incidence and risk factors of post-LAR RVF and to identify optimal treatment methods for patients with post-LAR RVF.

## RESULTS

### RVF incidence and time of occurrence

Among the 1,493 female patients analyzed, 24 were diagnosed with RVF, with an incidence rate of 1.61%. RVF occurred three to 1,161 days post-operation, with a median time of 19.5 days. (Table 1) shows the distribution of cases by time of RVF occurrence. Of note, RVF in our study was never caused by local recurrence of rectal cancer. Out of the 24 patients who underwent RVF treatment, six received neoadjuvant radiotherapy and only two received postoperative adjuvant radiotherapy. The time of RVF occurrence for the latter two patients was 21 days and 27 days after operation (prior to the start of adjuvant radiotherapy in both cases). Other patients in our study who received adjuvant radiotherapy didn't suffer from RVF. Five patients out of the 24 who presented RVF were diagnosed with distant metastases during follow-up, but no VEGF inhibitors were administered prior to RVF occurrence.

**Table 1:** Time of rectovaginal fistula occurrence

*n* = days after LAR	Number of patients with RVF	Percentage
1 < *n* ≤ 10	7	29.2%
10 < *n* ≤ 20	7	29.2%
20 < *n* ≤ 30	5	20.8%
*n* > 30*	5	20.8%

LAR = low anterior resection, RVF= rectovaginal fistula.

*In these 5 patients, RVF occured at 37, 90, 121, 274, and 1161 days after LAR.

### High-risk factors

The results of our analysis of possible RVF high-risk factors are shown in (Table 2). Univariate analysis showed that a short distance between the tumor and the anal verge, long surgery duration, unsatisfactory anastomosis, and heavy blood loss during surgery were significant high-risk factors. Furthermore, the risk factors with statistical significance in multivariate analysis were unsatisfactory anastomosis (HR 6.474, 95% confidence interval [CI] 2.236-18.743, *p* = 0.001) and the distance between the tumor and the anal verge (hazard ratio [HR] 0.650, 95% CI 0.496-0.851, *p* = 0.002). Results are shown in (Table 3).

**Table 2:** Univariate logistic regression analysis of risk factors

	RVF (+) *n* = 24	RVF (−) *n* = 1469	*p*-value
**Patient-related**			
Age (years)	54.4	57.2	0.24
Body mass index	23.7	22.9	0.18
Hypertension	5(21%)	294(20%)	0.89
Diabetes	1(4%)	88(6%)	0.66
History of weight loss	8(35%)	617(42%)	0.83
**Tumor-related**			
Distance between tumor and anus* (cm)	6.00	7.98	<0.001
Maximum tumor diameter (cm)	3.1	3.5	0.15
T stage			0.95
Tx	0	40	
T0 benign	0	75	
T1	2	136	
T2	9	310	
T3	5	372	
T4	8	536	
N stage			0.165
N0	16	847	
N1	7	375	
N2	1	247	
TNM stage			0.256
Benign or Tis	2	158	
I	6	247	
II	8	347	
III	8	605	
IV	0	112	
Lymph node dissected (mean)	14.1	13.9	0.871
Lymph node metastasis (mean)	1.04	1.72	0.350
Treatment-related			
Neoadjuvant radiotherapy	6 (25%)	176 (12%)	0.10
Surgery duration (minutes)	137.6	114.3	0.009
Laparoscopic surgery	2 (8%)	176 (12%)	0.63
Prophylactic ostomy	5 (21%)	147 (10%)	0.11
Unsatisfactory anastomosis	6 (25%)	59 (4%)	< 0.001
Intraoperative blood loss (ml)	115.0	64.4	0.002
Major combined organ resection	2 (8%)	59 (4%)	0.34
Combined resection of vaginal wall	1 (4%)	31 (2.1%)	0.499
Combined resection of uterus and ovary			0.390
None	22 (91.6%)	1398 (95.2%)	
Hysterectomy	0	1 (0.1%)	
Oophorectomy	1 (4.2%)	39 (2.7%)	
Hysterectomy+unilateral oophorectomy	0	5 (0.3%)	
Hysterectomy+bilateral oophorectomy	1 (4.2%)	26 (1.8%)	

*Distance between tumor and anus refers to distance from the lower tumor margin to the anus based on the results of digital anal examination and intraoperative measurement.

**Table 3:** Multivariate logistic regression analysis of high-risk factors

	*p*-value	Hazard Ratio	95% CI
Distance between tumor and anus	0.002	0.650	0.496–0.851
Surgery duration	0.19	1.006	0.997–1.014
Unsatisfactory anastomosis	0.001	6.474	2.236–18.743
Intraoperative blood loss	0.20	1.003	0.999–1.007

### Difference between patients who presented RVF within 30 days vs over 30 days after surgery

(Table 4) compares various characteristics of patients who presented RVF more than 30 days after LAR (20.8% of all patients) with those of patients who presented RVF within 30 days after LAR and the results are showed in. We found that patients who suffered RVF more than 30 days after LAR had a higher proportion of receiving prophylactic stoma (3/5 vs 2/19) and neoadjuvant chemoradiation(4/5 vs 2/19).

**Table 4:** Difference between patients who developed RVF within 30 days vs over 30 days after surgery

	RVF (≤ 30 days) *n* = 19	RVF (> 30 days) *n* = 5	*P* value (Univariate analysis)
**Patient-related**			
Age (years)	54.6	53.6	0.896
Body mass index	24.4	21.1	0.144
Hypertension	3 (16%)	2 (40%)	0.254
Diabetes	1 (5%)	0	0.619
**Tumor-related**			
Distance between tumor and anus (cm)	5.87	6.50	0.336
Maximum tumor diameter (cm)	3.96	4.70	0.349
T stage			0.79 (Chi-square test)
Tx	0	0	
T0 benign	2	0	
T1	6	1	
T2	5	2	
T3	6	2	
T4	0	0	
**Treatment-related**			
Neoadjuvant radiotherapy	2 (11%)	4 (80%)	0.001
Surgery duration (minutes)	132.6	156.4	0.234
Laparoscopic surgery	2 (11%)	0	0.471
Prophylactic ostomy	2 (11%)	3 (60%)	0.114
Unsatisfactory anastomosis	4 (21%)	2 (40%)	0.406
Intraoperative blood loss (ml)	128.8	68.0	0.564
Major combined organ resection	2 (19%)	0	0.471

### Treatment and prognosis

### Different treatment, follow-up and healing time

Surgeons may perform prophylactic stoma (colostomy or ileostomy) on patients at high risk of anastomotic leakage(AL) or RVF after LAR in order to divert feces. For patients with RVF, if a prophylactic stoma has been performed during surgery, there is no need for supplemental diversion after RVF occurs. On the other hand, most patients who have not undergone prophylactic stoma will receive supplemental diversion (colostomy or ileostomy). In our study, two patients received prophylactic stoma during the surgery. One case had a small RVF; thus, she opted for fistula suture instead of diverting stoma. The remaining 21 patients (87.5%) underwent colostomy or ileostomy as a supplemental diversion (including 18 cases of transverse colostomy alone, one case of transverse colostomy plus RVF suture, and two cases of terminal ileostomy alone). Supplemental diversion and prognosis statistics are presented in (Table 5).

**Table 5:** Supplemental diversion and prognosis statistics

Treatment method		Number of cases	Outcome description
Therapeutic ostomy	Transverse colostomy	18	Healed after ostomy (*n* = 14). Died before stoma closure due to tumor recurrence (*n* = 3). Not healed to date (*n* = 1).
	Transverse colostomy+RVF suture	1	Healed well
	terminal ileostomy	2	Not healed to date
Prophylactic ostomy	transverse colostomy or end ileostomy	2	Have not undergone stoma closure surgery to date
RVF suture without diverting stoma		1	Healed 1 month after surgery

After RVF occurrence, the follow-up time for patients ranged from 228 to 3,733 days (median = 1,774 days; mean = 1574 days). By the time we wrote the manuscript, 16 cases had healed, and this the healing rate was 66.7%(16/24). The approximate range of healing time was 30–1,225 days (median = 210 days; mean = 330 days).

Among the 21 patients who underwent therapeutic ostomy (supplemental diverting stoma), one patient who underwent transverse colostomy combined with RVF suture healed well. Another 14 patients also healed after ostomy surgery alone, without RVF suture. Three patients died before stoma closure surgery because of tumor recurrence, so their RVF healing status is unclear. By the time we wrote the manuscript, three patients with RVF had not yet healed. Therefore, the healing rate was at least 71.4% (15/21). Since most RVFs heal after colostomy or ileostomy, patients with RVF who received such surgeries may not need additional surgery such as muscle or tissue transfer flaps. The RVF healing time for the 21 patients who underwent supplemental diversion was ranged from 30 to 1,225 days (median = 222 days). Apart from the 21 patients, one patient underwent RVF suture alone instead of supplemental diverting stoma, healing one month after suture surgery. The two patients who underwent prophylactic ostomy had not yet undergone stoma closure surgery at the time of writing the manuscript.

Out of the 24 patients who suffered from RVF, six patients underwent neoadjuvant chemoradiation, four of which healed (67%) while two of them died before healing. There was no statistical association between the age of patients and the healing time of RVF(*P* = 0.928). Although our data suggested a correlation between healing time and age (i.e., the older the patient, the longer the time for RVF healing), the results were not statistical significant (Table 6).

**Table 6:** Comparison of healing time of RVF patients with different age

Mean healing time with different age		Comparison of mean healing time with different age		
Age	healing time (range)		healing time	*P* value
< 45	231 (189∼269)	< 45 vs ≥ 45	231 vs 352	0.574
45∼60	346 (111∼1225)	< 60 vs ≥ 60	382 vs 317	0.800
≥ 60	382 (30∼733)	< 45 vs ≥ 60	231 vs 382	0.742

### Distal colon irrigation

Some patients received distal colon irrigation during supplementary transverse colostomy. The process of distal colon irrigation was as follows: a large volume of saline was poured from the stoma into distal colon. The anus was dilated at the same time so that any residual feces in the distal colon could be washed out. This process continued until the saline discharged from the anus contained no feces.

Among the 19 patients who underwent therapeutic transverse colostomy (including 18 cases of transverse colostomy alone and one case of transverse colostomy plus RVF suture), eight received intraoperative distal colonic irrigation. Our statistical analyses revealed no significant difference in total hospitalization costs, hospitalization duration, or healing time between patients who received distal colonic irrigation and those who did not (Table 7).

**Table 7:** Length of hospitalization and cost with or without distal irrigation

Patients receiving therapeutic transverse colostomy		Distal irrigation	Without irrigation	*p*-value
With or without anastomotic leakage (*n* = 19)	Number of cases	8	11	
	Hospitalization costs*	9726	10829	0.516
	Days of hospitalization after ostomy (mean)	10.0	8.0	0.585
	Median healing time (days)	202	258	
	Mean healing time (days)	207	429	0.150
RVF only, without anastomotic leakage (*n* =14)	Number of cases	5	9	
	Hospitalization costs *	10217	10058	0.934
	Mean days of hospitalization after ostomy	5.4	8.4	0.268
	Median healing time (days)	184	283	
	Mean healing time (days)	184	462	0.384

^*^USD, cost of stoma closure is not included.

### Impact on hospitalization days and cost

The total number of days of hospitalization and postoperative hospitalization, and the total hospitalization cost, were significantly higher for the RVF group compared with the non-RVF group. The total cost of secondary hospitalization due to RVF treatment ranged between $1,722 and $4,148 USD (median = $2,384 USD; Table 8).

**Table 8:** Length of hospitalization and cost data

	RVF (+) *n* = 24	RVF (−) *n* = 2087	*p*-value
Days of hospitalization	27.0	18.0	0.005
Days of post-surgery hospitalization	20.8	12.2	0.008
Cost of hospitalization (USD)	8115	5982	0.003

## DISCUSSION

In this study, the incidence of post-LAR RVF was 1.61%, while previous publications reported incidence values ranging from 0.9 to 9.9% (Table 9). This difference may be due to inadequate number of cases recruited in previous studies and various inclusion criteria utilized by these studies. According to the literature, most anastomotic leakage(AL) occurs 3.5–8 days after LAR surgery [13–15]. In this study, RVF occurred 3–1161 days after surgery (median = 19.5 days). This indicates that RVF occurs later and over a broader time span compared with AL. The reason for RVF occurring later might be explained by the mechanism underlying the formation of RVF, such as local inflammation and the formation of abscess at the anastomosis site, which gradually causes edema of the vaginal wall, weakening the tissue and leading to eventual rupture and RVF. Another reason for RVF occurrence is stapling the vaginal wall during anastomosis in LAR surgery, which results in necrosis of the vaginal wall and RVF.

**Table 9:** Previously reported RVF incidence rates

Report	Number of RVF cases	Total number of cases	Incidence	Year of publication
Antonsen and Kronborg [7]	4	178	2.2%	1987
Baran et al. [3]	1	104	0.9%	1992
Nakagoe T et al. [5]	2	140	2.9%	1999
Kim et al. [4]	2	48	4.2%	2001
Kosugi et al. [1]	16	161	9.9%	2005
Matthiessen et al. [2]	20	390	5.1%	2010
Watanabe et al. [6]	11	371	3.0%	2015

High-risk factors for the occurrence of post-LAR RVF are unclear. However, our study here shows that the shorter the distance between the tumor and the anus, the greater the incidence of postoperative RVF. This is similar to previous studies showing that the incidence of colorectal AL increased in patients with tumors near the anus [16, 17]. The are two possible reasons for the distance between the anus and tumors being a predictor of RVF. First, the lower the location of the rectal tumor, the greater the area of the vaginal wound during rectum-vagina separation in LAR, leading to an increased probability of vaginal injury. Furthermore, lower tumor sites caused anastomosis at lower sites with poorer exposure, thereby increasing the probability of clipping the posterior vaginal wall with the stapler. There is also a greater probability of RVF occurrence in patients with a fragile and edematous bowel wall, poor anastomotic blood supply, obvious tension in the anastomosis site, or the presence of obstructive symptom before surgery. The surgeons will avoid stapling of the vagina at the time of colorectal anastomosis. However, it's difficult for the surgeons to perform stapling of the vagina under poor exposure conditions. Therefore, it is difficult to determine how many cases of RVF were secondary to stapling of the vagina. Surgeons can evaluate the status of the anastomosis site; *i.e.*, they can assess whether there's poor blood supply, obvious tension, fragile tissue, or edema at the anastomosis site, and perform an intraoperative air leak test [18, 19]; In this study, these factors were considered as unsatisfactory anastomosis. Both univariate and multivariate analyses showed that this factor was predictive of RVF occurrence.

Univariate analysis identified long surgery duration and heavy intraoperative bleeding as high-risk factors for RVF, but multivariate analysis did not identify these as independent factors.

We also investigated whether neoadjuvant radiotherapy increases the risk of RVF. Radiotherapy may lead to vascular injury, chronic inflammation and ischemia, which may lead to RVF formation [20]. Previous studies have reported that neoadjuvant radiotherapy has no influence on the incidence of AL [21]. The results of our study showed that neoadjuvant radiotherapy was not a high-risk factor for RVF.

A variety of methods and materials have been reported for vaginal repair and RVF treatment [12, 24–26]. Early intervention including supplementary diverting stoma can improve the success rate of RVF healing [22]. Diverting stoma can also reduce the pressure gradient between the rectum and the vagina [23], which helps fistula heal after repair surgery. Out of the 24 patients who suffered from RVF in our study, 21 patients underwent colostomy or ileostomy as a supplemental diversion after RVF occurrence (including one case receiving RVF suture at the time of colostomy). The patients who underwent supplemental diversion didn't receive fistula-repair surgery (*i.e.*, muscle or tissue transfer flaps). 16 patients were confirmed to have healed from RVF during follow up. Thus, we found that most cases of RVF can heal after colostomy or ileostomy. Therefore, patients with RVF may not need additional surgery such as muscle or tissue transfer flaps.

Distal irrigating during surgery in patients undergoing transverse colostomy or ileostomy can theoretically promote early fistula healing or shorten hospitalization time. However, we found no significant difference between the irrigation and non-irrigation groups in our study in terms of total hospitalization cost and time.

The role of prophylactic stoma in the prevention of postoperative RVF is unclear. In a clinical trial involving 234 patients, Matthiessen *et al.* reported that prophylactic stoma can reduce the incidence of AL, but its impact on RVF is unclear [27]. Theoretically, prophylactic stoma can reduce the incidence of RVF. However, ostomy itself has a negative impact on patients [28, 29]. Patients who undergo intraoperative prophylactic ostomy often present low tumor location, tissue adhesion, bowel edema, obstruction, and other risk factors. If intraoperative prophylactic ostomy is not performed, these patients may have higher RVF incidence and poorer prognosis. However, the prognosis of patients receiving prophylactic stoma is not comparable with that of patients who do not undergo prophylactic ostomy. In this study, we found no correlation between prophylactic ostomy and RVF occurrence. Therefore, the value of prophylactic ostomy for RVF remains controversial, and a prospective randomized controlled trial is warranted to further investigate this issue.

As RVF may be secondary to AL, it is not easy to form a drainage system. From our experience, we recommend puncture and other measures to open drainage in patients with AL and local abscess. Expansion of the rectal fistula can also facilitate drainage.

RVF occurrence significantly increases the duration and cost of hospitalization, with an average increase of 10 days in hospitalization, which does not include subsequent hospitalization for RVF treatments. This is similar to the increase of 7.3 days in the duration of hospitalization for gastrointestinal AL reported previously in the literature [30]. A previous study reported that gastrointestinal AL results in an additional cost of $24,129 USD [30]. RVF occurrence has a serious impact on the quality of life of patients and increases their psychological burden. Many patients have long-term or even lifelong stoma bags, which can severely decrease their quality of life.

Our study suffered from some limitations. For example, this was a single-center retrospective study. Furthermore, we did not analyze the nutrition/malnutrition status of patients after LAR surgery. Moreover, the follow-up time for some patients was only six months. Despite these limitations, our study introduces novel analyses compared with previous publications [2, 6]. For example, our sample size was much larger than that of previous publications. In addition, our study is the first to address RVF after LAR among Chinese patients. Furthermore, we found that a short distance between the tumor and the anus, as well as unsatisfactory anastomosis, are a risk factors for RVF. Importantly, we didn't find any correlation between neoadjuvant chemoradiation and RVF. The incidence of RVF was 1.61%. Lastly, we identified supplementary diverting stoma as a reasonable and effective treatment for post-LAR RVF. This information may help doctors to choose optimal treatment strategies for post-LAR RVF in clinical settings.

## MATERIALS AND METHODS

### Patients

**Figure 1:**
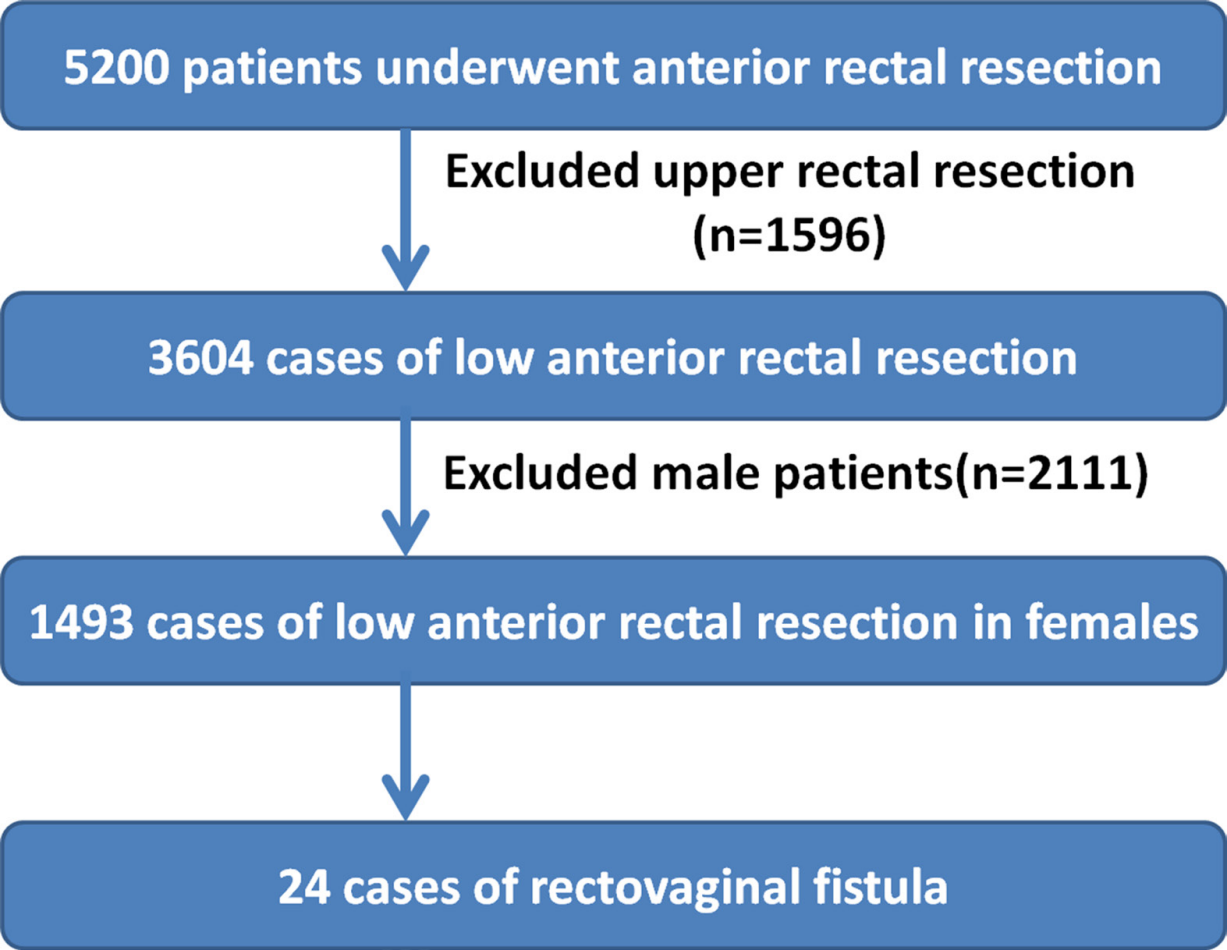
Work Flow Chart Exclusions from 5200 patients who had a resection between January 2006 and March 2016.

A total of 5,200 patients with colorectal cancer who underwent LAR at Fudan University Shanghai Cancer Center between January 2006 and March 2016 were enrolled in this study. In 3,604 of these patients (1,493 women, average age 57.2±11.9 years; range 22–93 years), the distance between the lower edge of the tumor and the anal verge was under 12 cm. We performed detailed analyses on the 1,493 female patients. Laparotomy was performed on 1,322 patients and laparoscopic surgery on 171 patients. All patients received intraoperative circular-stapled anastomosis, with no patient receiving hand-sewn coloanal anastomoses. The date of last follow-up was September 30, 2016, and all patients were followed-up for at least six months. The recruiting process of our study is shown in (Figure 1).

### Criteria for diagnosis and evaluation of RVF healing

RVF was diagnosed by clinical symptoms that indicate communication between the vagina and the rectum, such as feces, gas or intestinal fluid discharged from the vagina, and methylene blue solution flowing out of the vagina after it was injected into the patient's anus. RVF was confirmed by rectal and gynecological examination, and by endoscopic or radiological investigations.

The healing criteria for RVF are as follows: 1. No feces or gas discharged from the vagina. 2. Confirmation by a surgeon that the RVF has been healed, after performing vaginal and rectal palpation; 3. No methylene blue solution should flow out of the vagina after injecting it into the patient's anus and a applying pressure to the rectum,. 4. No RVF can be found through colonoscopy.

### Data analysis

SPSS 22.0.0 software was used for data analysis. We performed univariate and multivariate logistic regression analyses to identify high-risk factors of RVF after LAR surgery. The variables used for univariate analysis were age, body mass index, hypertension, diabetes, history of weight loss, neoadjuvant radiotherapy, surgery duration, surgical method (laparoscopic surgery or laparotomy), methods of intraoperative prophylactic ostomy (transverse colostomy or ileostomy), unsatisfactory anastomosis (the definition including any of the following five criteria: poor blood supply for anastomosis site, obvious tension in anastomosis site, fragile tissue in anastomosis site, obvious edema in anastomosis site, and positive intraoperative air leak test), intraoperative blood loss, combined organ resection, distance between the lower edge of the tumor and the anal verge, maximal tumor diameter, T stage, days of hospitalization, days before surgery, and total hospitalization costs. Factors that showed statistical significance (*p* < 0.05) in univariate analysis were subjected to multivariate logistic regression analysis. *P*-values less than 0.05 were considered statistically significant.
